# The DSMZ Digital Diversity Annotation Hub: a pipeline for database expansion via text mining and human curation

**DOI:** 10.1515/jib-2025-0058

**Published:** 2026-08-17

**Authors:** Emanuel Quadros, Lorenz C. Reimer, Julia Koblitz

**Affiliations:** Leibniz Institute DSMZ-German Collection of Microorganisms and Cell Cultures, Braunschweig, Germany

**Keywords:** database expansion, data annotation, text mining, relation extraction, named-entity recognition

## Abstract

Maintaining scientific databases that depend on continuous curation of research literature often requires labor-intensive, slow, and error-prone annotation processes. To address these challenges, we present a pipeline that integrates text mining with expert supervision to support database expansion. Using the BRENDA enzyme database as a case study, we compiled a relation extraction dataset by aligning document-level annotations with literature references through distant supervision. We then developed a neural model that performs entity recognition and relation classification, enabling the extraction of enzyme-strain associations from full-text articles. To close the loop between machine learning and expert curation, we designed a web-based interface that allows annotators to review and refine predicted relations. While preliminary, our initial experiments show the potential of combining weak supervision and human-in-the-loop validation to accelerate the integration of literature-derived information into knowledge bases.

## 1 Introduction

Scientific databases provide a valuable window into the wealth of information scattered in the research literature by consolidating it into a curated structure and a unified interface. However, compiling this information and systematically annotating it is a time-consuming effort, often requiring the involvement of numerous annotators and, consequently, the transmission of skills and annotation standards through the lifespan of the project. Those demands impose not only heavy budget requirements but also the risk of the accumulation of errors in long-living projects. On top of that, the human resources available to annotation projects can hardly match the speed at which the research literature grows, leading to delays in the incorporation of new findings in the databases or large gaps in their coverage, as curators focus on narrower, more manageable areas of their subject areas. The curation of literature resources for the maintenance and expansion of scientific databases is, therefore, an area that is ripe for automation, one where we can ease the labor of curators and annotators while improving the quality and the speed with which structured data becomes available to the public. We report here on the development of a complete pipeline for database expansion, including the compilation of a machine learning dataset from the existing structure of a database, the development of a suitable text mining model, and the creation of a tool to streamline the process of computer-assisted curation by domain experts.

As our initial focus, we chose a domain of particular importance for strain discovery in research and bioindustry applications, namely the availability of metabolic information at the strain level in the BRENDA enzyme database [1], 2]. BRENDA is a large collection of functional enzyme and metabolic data established in 1987 and selected as an ELIXIR Core Data Resource in 2018, counting data points for 112,288 enzymes and 15,335 associated organisms. Part of the richness of BRENDA comes from its more than 173,164 literature references, from which a vast amount of enzymatic data has been collected over the years, including on the relation between enzymes and specific organisms. In most cases, however, this association has been made at the species level, which limits the usefulness of the database for applications in which screening for specific strains for the production of an enzyme, for example, is crucial. Therefore, we go back to the literature in this work, in order to extract more fine-grained metabolic information on bacterial strains by the use of natural language processing techniques.

This task involves two natural language processing subtasks: named-entity recognition (NER) and relation extraction (RE). The first task refers to the identification of terms in a text that refer to entities in a particular domain and to their classification into a set of categories. In (1), for example, identifying “branched-chain amino acid aminotransferase” as an enzyme, “*Mycobacterium tuberculosis*” as a bacterial species, and “H37Rv” as a bacterial strain. The second task, relation extraction, refers to the semantic classification of the relations between entities in a text. In this case, the task would involve recognizing that “H37Rv” is a strain of “*M. tuberculosis*”, and that this strain can express “branched-chain amino acid aminotransferase”.

(1) “The gene encoding for branched-chain amino acid aminotransferase in *M. tuberculosis* H37Rv has been cloned, expressed, and characterised.” [3].

In the following section, we briefly comment on related text mining and annotation projects. Next, we describe the challenges involved in compiling an appropriate dataset for named-entity recognition and relation extraction, especially in the absence of preannotated data at the token level, and our approach to this problem. We then discuss the development of a model capable of recognizing entities in text and the relations between them. Finally, we report on the development of an annotation tool powered by the predictions of this machine learning model.

## 2 Related work

A number of projects have been undertaken to assist literature annotation with computational tools. Important examples we will briefly mention here are TeamTat [4], AIONER [5], and PubTator 3.0 [6]. Taken together, these tools form a pipeline of sorts, in that TeamTat has been used in the annotation of NLM-Chem [7] and NLM-Gene [8], which figure among the corpora used to train AIONER. This model, in turn, provides automatic annotations to PubTator 3.0.

TeamTat is a feature-rich annotation tool with project management features to facilitate collaboration within a team of annotators. Unlike other annotation tools, it also provides relation annotation capabilities and allows the use of custom ontologies. Although we take inspiration from TeamTat, we choose to develop a new annotation interface to facilitate the development of a modern interface and new features, such as seamless integration with text mining predictions, and a review interface for curators that allows the evaluation of proposed relations across multiple annotated articles.

On the text mining side, the closest comparison to our model is AIONER, an entity recognition model trained on publicly available datasets focusing on six entity types: gene, disease, chemical, species, variant, and cell line. The model achieves very good results on these categories by merging a diverse collection of individually small but collectively significant biomedical entity recognition datasets containing annotations at the sentence level. This was done in order to mitigate the scarcity of quality annotations, which is a common challenge in the development of text mining models. We take a different approach here by relying on document-level annotations, which makes the task computationally more challenging but allows us to include documents that are annotated simply with respect to whether particular entities and relations occur in the text, without requiring expensive manual sentence-level annotations. Moreover, as described below, we add relation extraction to the same model, again relying on document-level annotations.

The annotations made by AIONER are mapped to entity identifiers and added to PubTator 3.0, which is a web-based tool that allows users to search for mentions of those entities in PubMed abstracts and PMC articles processed by the model. We take inspiration from this tool in developing the curation part of our annotation platform. While PubTator 3.0 is tailored to literature searches in the PubMed database that are focused on a particular entity or relation, our tool provides curators with an overview of annotations made by different annotators, with respect to a particular entity or relation, across the literature references within an annotation project. The goal is to allow curators to efficiently evaluate them for inclusion in their database.

Text mining has also been used to assist literature annotation within the BRENDA ecosystem, where the tools FRENDA and AMENDA have been used to screen the literature for organism-specific enzyme information [9]. FRENDA uses dictionaries of enzyme and organism names, along with synonyms and spelling variants, to find articles where enzyme and organism names co-occur in the PubMed database. AMENDA then extracts a subset of those references by using a ranking system designed to find the most relevant co-occurrences. In addition, AMENDA adds information about the subcellular localization of enzymes and the source tissues in which they are active, again by matching articles against a dictionary of terms. While these tools have facilitated the expansion of the BRENDA database over the years, they rely on dictionary matching, which entails a restriction to the list of terms already known, the need to exclude ambiguous names, and the maintenance of spelling variants. Accordingly, FRENDA shows a large number of false positives, and AMENDA a high miss rate [9]. More importantly, adapting these methods to the annotation of other database fields would be impractical, as each field would require the maintenance of a new dictionary of terms. The work reported here is intended to maintain the strengths of the text mining approach that has already been used in BRENDA, while gaining in accuracy and generalizability.

## 3 Dataset compilation

A significant challenge in relation extraction is the lack of explicitly annotated texts, particularly in specialized domains. This means that supervised learning approaches, which rely on labeled datasets, are often unfeasible. When we have a high-quality knowledge base at hand, however, this challenge can be circumvented through *distant* (or *weak*) supervision, whereby the texts forming the training data are aligned to the knowledge base. Since in the ideal case we would like to have annotations at the sentence level, the alignment between texts and the knowledge base is often expressed through the assumption that if a knowledge base contains a relation between two terms, then sentences containing those terms express that relation [10]. For example, sentences (2) and (3), occurring in the same article, would both be tagged as expressing the relation hasenzyme between ATCC 25544 and cholesterol oxidase (COX), even though only the first one actually supports this relation. While this tagging would provide us with more training samples, since each sentence containing the entities in question would count as support, it also risks leading to high false negative rates, as the model would take contexts like (3) as providing evidence for the purported relation.

(2) “The amount of extracellular **COX** of the non-pathogenic strain *R. erythropolis* **ATCC 25544** obtained in a 1.5 1 batch fermentation represents a 36 % of the activity (130 out of 360 U/mL) when measured directly from the broth, but after the 6 % Triton X-114 treatment and phase separation it represents a 65 % of the total activity” (boldface added) [11].
(3) “The suitability of the strain ***Rhodococcus erythropolis* ATCC 25544** grown in a two-L fermentor as a source of **cholesterol oxidase** has been investigated.” (boldface added) [11].

We employ here a weaker version of this heuristic to align training texts with the knowledge base, namely, “if two entities participate in a relation, **at least one sentence** that mentions these two entities might express that relation.” [12]. Applied to the document-level annotations in BRENDA, we can infer from this assumption that if the database states that a given relation between two entities is supported by a document, at least one of the joint mentions of said entities in the document expresses that relation – in the previous example, sentence (2). If we can work under this assumption, it is sufficient to have a dataset with annotations at the document level, stating for each document the relations that are present in it. The modeling challenge imposed by this kind of annotation, however, is that we would like to be able to locate where in a text a relation extracted by the model receives support (in (2), not in (3)) and, at the same time, we want to make sure that the model learns from the sentences that support the presence of a relation when it is in fact present, even if other sentences in the same document mention the entities in question but do not support a relation between them. In Section 4.3, we discuss how pair representations are pooled in order to solve this issue.

Once we have a dataset with relations explicitly marked, we still face the issue that negative examples are necessary for the training of discriminative machine learning models. In contrast, most corpora annotate *only* true relations and omit explicit marking of unlinked pairs, since it would be extremely costly to annotate all pairwise combinations. Here, again, having a high-quality knowledge base at our disposal is advantageous. By leveraging the manual curation in BRENDA under a closed-world assumption [13]: whenever a document is annotated as containing two entities but a relation between them has not been annotated by the database curators, we infer a negative label for that pair.

The resulting dataset includes the 11,399 BRENDA references that are available in full text via PubMed Central, excluding those that are annotated as mentioning bacteria but not a bacterial strain. The reason for this exclusion is that those are precisely the articles whose annotation this project aims to expand, that is, those for which BRENDA has information down only to the species level. Moreover, if these articles were kept in the dataset used to train the model, they would count as data samples that do not contain any bacterial strain or strain-enzyme relation, regardless of whether the text actually mentions a bacterial strain or supports a relation between it and an enzyme.

**Figure 1:**
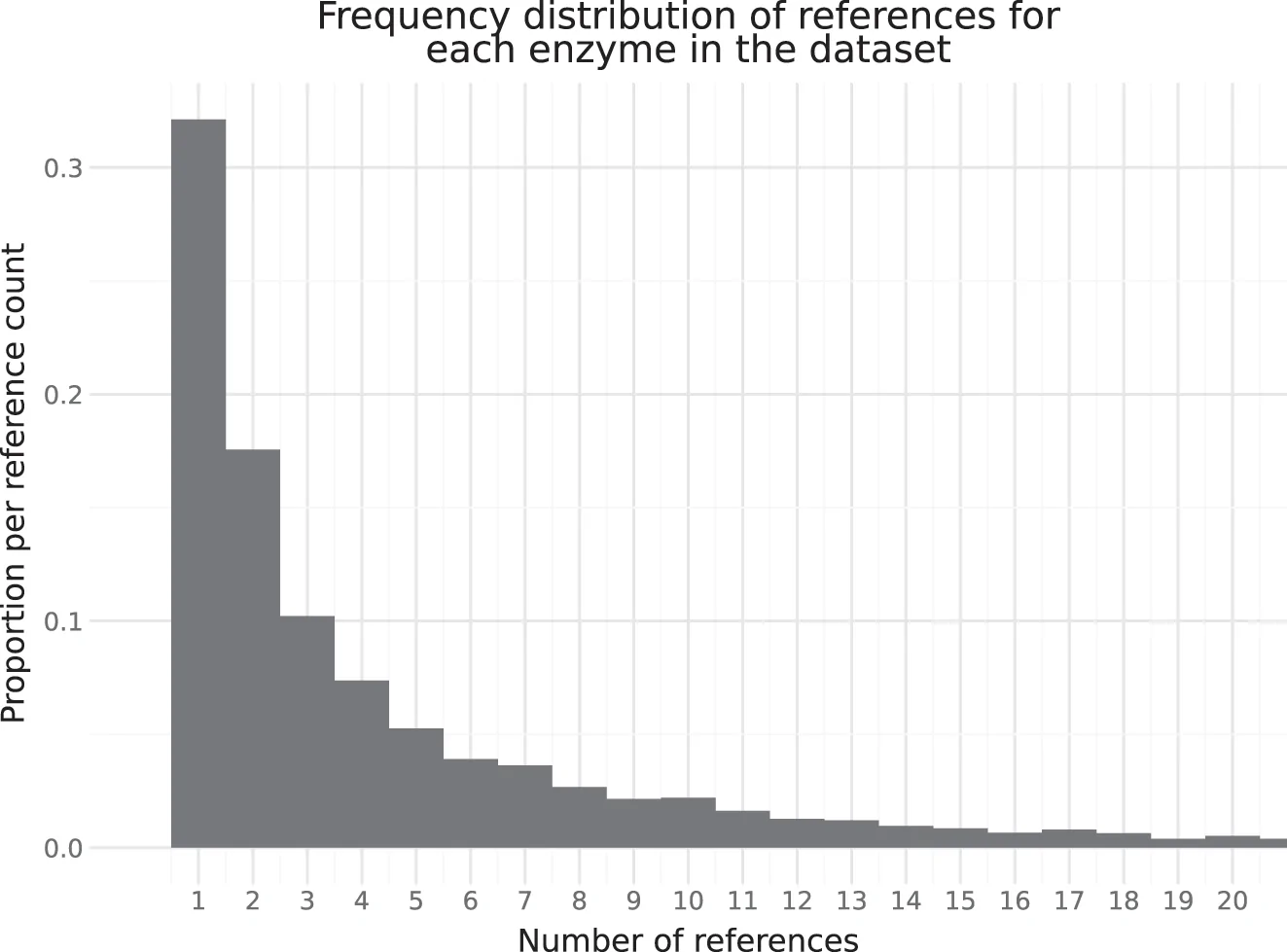
Frequency distribution of reference counts for each enzyme. The plot shows reference counts up to 20.

The distribution of references mentioning entities within each entity type is highly imbalanced in this dataset. Illustrating this, many enzymes are only referenced by one document in the full text dataset (Figure 1), but there is a large range of reference counts across the set of enzymes. Table 1 displays reference count statistics for each entity type considered here and for strain–enzyme relations. For those items that are referenced multiple times, we want to make sure that documents containing them are well represented in the training data, as well as in the validation and test data.

**Table 1:** Number of bacterial species, strains, enzymes and strain–enzyme relations in the full text dataset, along with the mean and range of reference counts per category.

Entity	*n*	Reference counts	
		Mean	Range
Bacteria	1,307	3.84	[1, 671]
Strains	2,663	1.78	[1, 309]
Enzymes	3,636	3.91	[1, 75]
Strain–enzyme relations	13,977	1.36	[1, 61]

In order to ensure that our training (70 %), validation (15 %), and test (15 %) data splits are sufficiently diverse with respect to the set of entities mentioned in each of them, we employ a greedy maximum entropy sampling strategy [14], where documents are added to a split sequentially by selecting, at each step, the one that maximizes Shannon’s entropy over the probability distribution of organisms in that split.

A final challenge in the compilation of the dataset is specific to the goals of BRENDA. We have seen that references are annotated at the document level, indicating what entity classes (bacteria, strain, enzyme, or other organism) and which specific entities belonging to those classes are mentioned in the text. However, since the database collects only references that relate organisms to enzymes, all the articles in the database mention at least one enzyme. Therefore, the dataset extracted from BRENDA does not, by itself, contain references serving as negative examples for the enzyme class. To mitigate this issue, we include in the training set 850 papers from the field of psycholinguistics that are also available in full text via PubMed Central and are virtually guaranteed not to contain enzyme mentions. The validation and test splits also received, respectively, 100 and 50 psycholinguistics papers.

Having described how the dataset was constructed, we now turn to the design of the model architecture and the learning setup.

## 4 Approach

**Figure 2:**
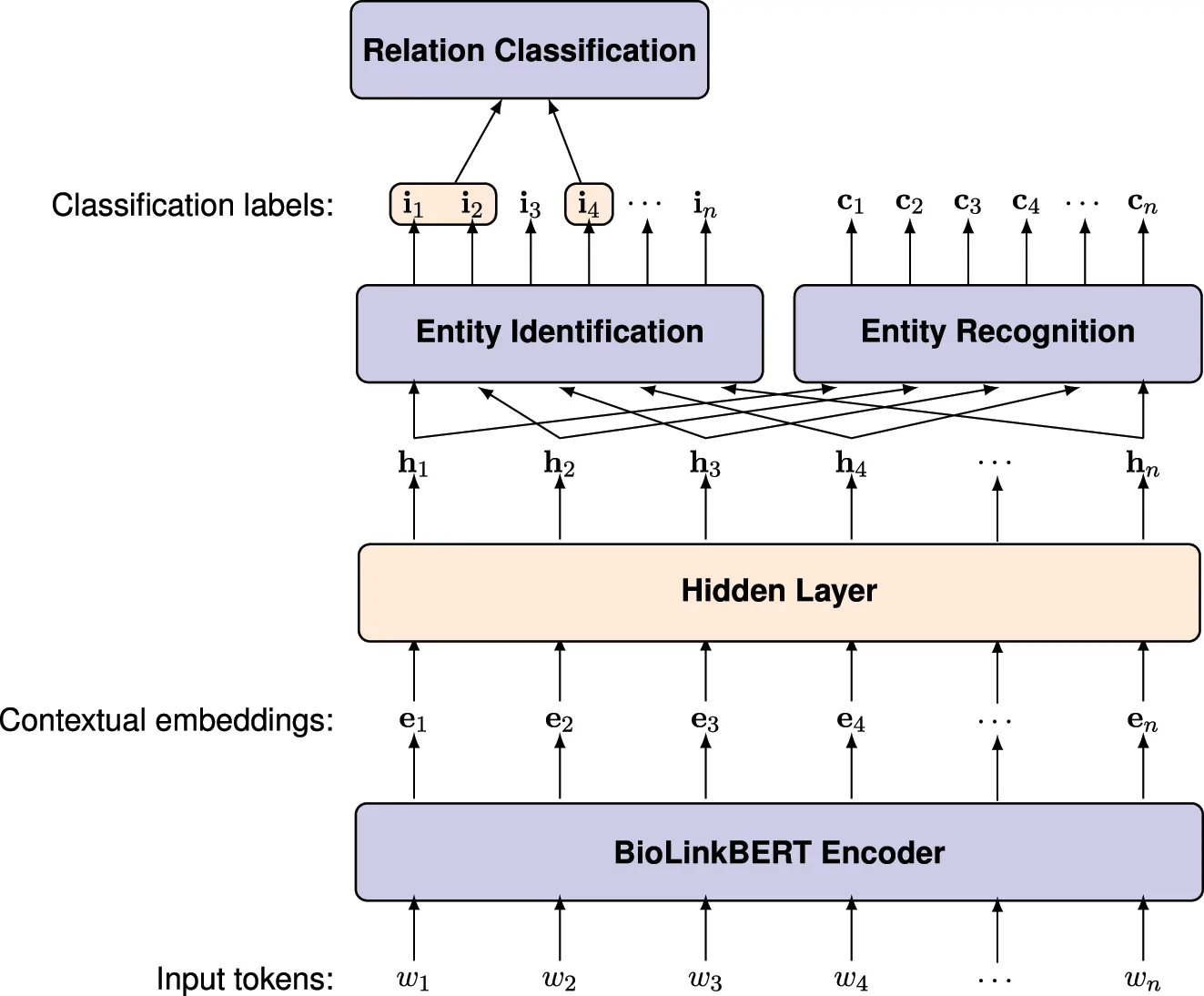
Overall architecture of the proposed joint model for entity recognition and relation extraction. Given an input sequence of tokens, a transformer encoder, namely BioLinkBERT [15], produces contextual embeddings that are further processed by a hidden layer and passed to two parallel modules: (i) the *entity identification head*, which predicts document-level identifiers *i*_1_, …, *i*_*n*_ by pooling token-level logits with LogSumExp; and (ii) the *entity recognition head*, which assigns class labels *c*_1_, …, *c*_*n*_ using the same pooling strategy. At inference time, tokens whose maximum entity probability exceeds a learned threshold are retained as entity mentions, while other tokens are implicitly treated as none. Detected entities are then grouped into candidate pairs, and their embeddings are aggregated (via either thresholded “hard” representations or attention-weighted “soft” representations during training). Each pair is classified by a biaffine relation classifier into one of hasenzyme, hasspecies, or none, with multiple mentions of the same entity pooled by LogSumExp to yield document-level relation predictions.

We treat entity recognition and relation extraction as joint tasks hierarchically organized, such that the token-level representations learned for entity prediction form the basis for relation classification, as shown in Figure 2. The figure outlines the step-by-step processing of input word tokens *w*_1_…*w*_*n*_ into numerical contextual embeddings *e*_1_…*e*_*n*_ and into intermediate representations, *h*_1_…*h*_*n*_, generated by neural network layers trained on the present task. These representations are passed to the entity recognition classifier, which generates a sequence of category labels, *c*_1_…*c*_*n*_, one for each token, and to the entity identification classifier, which generates a sequence of entity labels, *i*_1_…*i*_*n*_, one for each token. Token representations that receive a valid entity label are then sent to the relation classifier, responsible for predicting which pairs of entities are related by one of the relations of interest.

This setup allows improvements in entity recognition to propagate upward to relation extraction, while relation supervision provides additional learning signals that shape lower-level representations. This design combines the advantages of shared representations across subtasks [16] and a sequential architecture where entity recognition feeds relation extraction [17]. As described in the next section, the token representations used for entity classification are extracted from the last layer of BioLinkBERT. The source code of the model is available at https://github.com/manuquadros/d3text/releases/tag/v0.1.0, and the accompanying data processing pipeline can be found at https://github.com/manuquadros/brenda_references/releases/tag/v0.1.0.

An alternative approach to this task would be zero-shot classification, where predictions for unlabeled data are generated by prompting an LLM without its having been specifically trained on the classification task at hand. One could call it, informally, the “Just ask ChatGPT” approach. Three main considerations argue against this alternative and for the development of custom classifiers on top of a relatively small LLM like BioLinkBERT. First, zero-shot classifiers have shown poor results in named-entity recognition, particularly in specialized domains, even when powerful LLMs like ChatGPT-4 are used [18], and even with the addition of careful prompting techniques, such as the inclusion of entity type descriptions [19], or in multi-agent setups [20]. Second, prompt-based strategies are known to be brittle (highly sensitive to details of the query) and inconsistent (even when the prompt is held constant) [21]. Finally, energy use, both during training and during inference, is highly correlated with model size [22]. Training GPT-4, with 1,800 billion parameters, consumes at least 34.5 thousand times more energy and generates at least 17.8 thousand times more CO_2_ emissions than training a BERT-like model, with its 0.1 billion parameters [23]. Therefore, since custom=trained lightweight classifiers are more reliable for specialized classification tasks and have a considerably smaller environmental impact, they ought to be the first-line choice for NER and RE tasks.

### 4.1 Feature extraction

Given a sequence of input tokens *w*_1_, *w*_2_, …, *w*_*n*_, the model produces contextualized embeddings via a transformer encoder and subsequent hidden layers. We use BioLinkBERT [15], which is an extension of the BERT [24] framework. The capability of BERT models to extract relevant features for token classification comes from its training regime, consisting usually of two tasks: masked language modeling and next sentence prediction. The first one involves masking a portion of the tokens in a sequence of text and predicting those masked tokens from the representation of the non-masked tokens. In the second one, the model uses the representation of the tokens in sentence A to predict whether sentence B follows sentence A in the dataset or if it is a sentence randomly sampled from another portion of the dataset.

BioLinkBERT builds on BERT by including contextual information beyond the same document. The model is also trained on masked language modeling but replaces next sentence prediction with document relation prediction, where two segments of text A and B (typically sentences or paragraphs) are classified according to whether (i) B follows A in the same document, (ii) B is in a document linked to A’s document (either through hyperlinks or reference citations), or (iii) B is randomly selected from a document not linked to A’s document. As of the time of this publication, BioLinkBERT has stood at the top of the BLURB leaderboard, which compares BERT-derived language models on biomedical tasks, including NER and RE [25]. BioLinkBERT has also shown better results when compared to other LLMs (namely, GPT-3, BioGPT, BioMedLM, BERT, BioMegatron, PubMedBERT, and BioClinicalBERT) in the prediction of microbiome-disease relationships [26].

### 4.2 Entity identification and classification

From the token embeddings described in the previous subsection, two parallel classification heads predict:

– **Entity identifiers** *i*_1_, *i*_2_, …, *i*_*n*_ drawn from the set of entity identifiers defined in the knowledge base, plus a special unknown label. These are selected by a classifier module applied to each token embedding.
– **Entity class labels** *c*_1_, *c*_2_, …, *c*_*n*_ drawn from the set {bacteria, strain, enzyme, other organism, out-of-scope}. These are generated by another classifier module, parallel to the identifier head, aiming to allocate entity expressions into their major classes, even when they cannot be assigned to an entity identifier present in the knowledge base.

Since our gold labels are defined at the document level rather than the token level, token logits are pooled across the sequence to produce document-level predictions for each label. We use a LogSumExp pooling strategy [27]: for each entity or class dimension, we compute

(1) $$z_{k}=\log\overset{n}{\underset{t=1}{\sum }}\exp\left(z_{t,k},k\right)$$

where *z*_*t*,*k*_ is the logit for class *k* at token *t*. This pooling strategy has the effect of approximating a maximum function over tokens while being differentiable, thus retaining smooth gradients and ensuring that a single strongly predictive token is sufficient to activate the document-level label. These pooled logits are then trained against the document-level multi-label targets using binary cross-entropy, with label smoothing.

At inference time, entity detection is made more selective by thresholding the maximum sigmoid probability across entity classes for each token. Tokens whose maximum probability exceeds a learned threshold are marked as candidate entity mentions, and their token embeddings are retained for relation classification. This mechanism provides an implicit “none” outcome for tokens that do not belong to any entity.

### 4.3 Relation classification

Relation extraction is defined at the entity-pair level. For each candidate entity pair (*i*, *j*) within a document, the model computes a relation label *r* ∈ {hasenzyme, hasspecies, none}. To construct entity pair representations, we aggregate the contextualized embeddings of the selected entity tokens:

– **Hard representations:** for detected entities (those surpassing the threshold), we directly use their token embeddings.
– **Soft representations:** for gold entities (during training), we compute an attention-weighted sum of token embeddings, where the weights are proportional to the entity classifier’s logits for that entity class. This allows relation supervision to flow even when entity detection is uncertain, ensuring that all gold relations contribute a loss signal.

Entity representations *x* and *y* are then fed into a biaffine relation classifier. This module applies both a bilinear interaction of the form *x*^⊺^*W*_*r*_*y* for each relation type *r* and a linear transformation over the concatenated pair [*x*; *y*]. The two terms, plus a learned bias, are summed to produce relation logits. Training uses cross-entropy with a designated none label so that non-related pairs are explicitly modeled as negatives.

To handle multiple mentions of the same entity identifier within a document, relation logits are pooled across mentions using LogSumExp, similarly to what we described in eq. (1). This strategy yields a single prediction per entity pair in a document. In this way, we address the desiderata laid out in Section 3, namely, (i) we derive logits for pairs of entity term mentions, which allows us to locate the sentences in a document that provide the most evidence for a relation,1In our tokenization procedure, we are careful to record the position of each token in the text, which allows us to retrieve the sentential context in which they appear. and (ii), by pooling predictions through LogSumExp, we derive a final prediction for the entity pair that is most strongly influenced by the entity mentions that provide the most support for each relation type.

### 4.4 Results

Table 2 summarizes the performance of the model on entity recognition. On this task, which involves predicting a class label for entities present in the document, the model achieves a weighted F1 score of 0.90, with high precision and recall across all categories. This shows that the model is able to reliably allocate mentions into broad semantic classes. We observed lower recall for strain and lower precision for bacteria. Since terms for bacterial species and for bacterial strains refer to strongly related conceptual entities, appearing, therefore, in similar textual contexts, we can speculate that some strain-annotated terms are predicted by the model to refer to bacterial species, hurting the recall score of the former and the precision score of the latter.

**Table 2:** Entity recognition (class prediction) results.

Class	Precision	Recall	F1	Support
Strain	0.76	0.66	0.71	419
Bacteria	0.69	0.90	0.78	267
Other organism	0.95	0.88	0.91	819
Enzyme	1.00	0.98	0.99	1,174
Micro average	0.91	0.89	0.90	2,679
Macro average	0.85	0.85	0.85	2,679
Weighted average	0.92	0.89	0.90	2,679

While the results for entity recognition are encouraging, those for entity *linking* (that is, for the more challenging task of predicting the correct identifier from the universe of 7,606 possible entities) were unsatisfactory. In this case, the model did not reach meaningful results, with F1 scores close to zero, as shown in Table 3. This outcome is not unexpected: the task corresponds to an *extreme multi-label classification* problem in which most identifiers are very rare, documents contain only a handful of positives, and negative evidence is inferred only indirectly. The more encouraging LRAP score (0.31) shows, however, that the model is often able to assign higher ranks to the correct entities, suggesting that re-ranking or candidate-pruning strategies may substantially improve the precision of the model in the future.

**Table 3:** Entity identifier prediction results over 6,861 possible identifiers.

Metric	Score
Micro-F1	0.06
LRAP	0.313

Given that relation extraction is hierarchically dependent on entity identification in the current architecture of the model, the F1 scores are similarly close to zero. We interpret these results as a demonstration of the difficulty of document-level entity linking in this domain. Since class-level classification is a more stable intermediate target, as it involves fewer classification labels, each with more training data points, a clear direction for future work is using class information to reduce the set of candidates for entity linking to those terms that received a class-level label.

In the following section, we report on the development of our data annotation interface, presenting examples from the reasonably successful entity recognition component of the model, as well as one of the examples of correct relation classification generated at this stage.

## 5 Data curation

While the model forms the core of the pipeline, its ultimate goal is to support the expansion of biological databases with high-quality data. Therefore, we still need a step of expert human supervision to make sure that the predictions made by the model meet the standards of the database. In line with this goal, we have developed the prototype of a web interface to facilitate the workflow of annotators, as part of the DSMZ Digital Diversity Annotation Hub. This interface is currently in heavy development and testing at the DSMZ.

**Figure 3:**
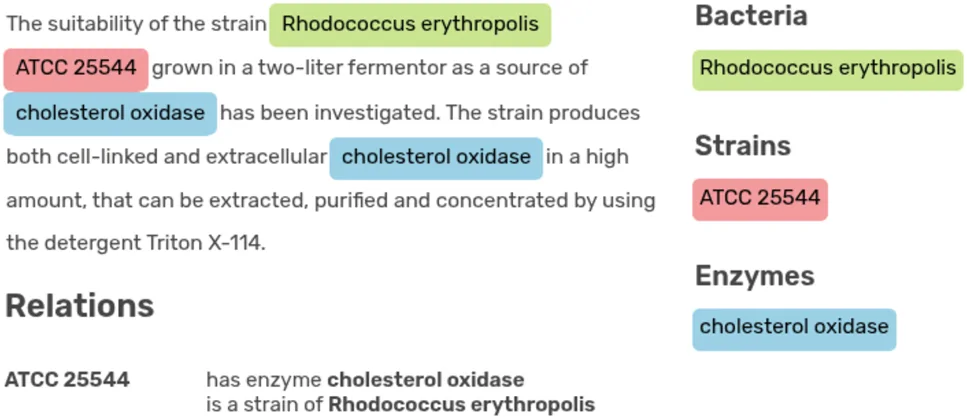
The annotation interface in its annotation mode.

The annotation hub operates in two modes: annotation and review. In the annotation mode (Figure 3), users are presented with an interface augmented with predictions made by the model described in the previous sections. Annotators may reject or edit these predictions, as well as add new annotations.

**Figure 4:**
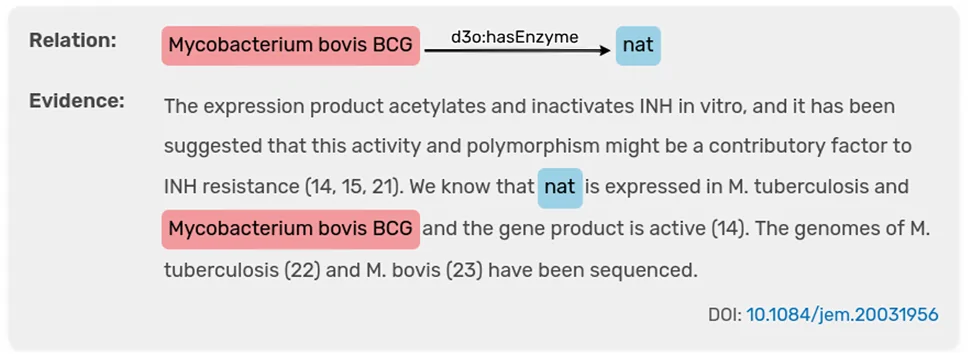
The annotation interface in its review mode.

In the review mode (Figure 4), the focus shifts to the evaluation of particular relation triples as candidates for inclusion in the database. The interface presents excerpts of text predicted by the model to provide support for a relation triple, and the annotator can edit them, reject them entirely, or accept the labeling of the entities as correct while rejecting them as offering support for the relation in question.

Importantly, given its focus on relation triples across the sample and not on individual articles, the review mode can present excerpts from different articles simultaneously so that curators are provided with an overview of the support that a relation finds in a sample of literature references. For example, in Figure 4, we have evidence for a relation between *Mycobacterium bovis* BCG and *N*-Acetyltransferase (NAT), but in this particular excerpt, crucially, the evidence is only established indirectly, through a citation. By having quick access to other excerpts supporting this relation in the same and in other articles, the curator is able to assess how strongly the relation is supported across the sample and whether other articles, such as the one cited in this example, should be included in the sample of references. Improvements to this curation view that are currently in development include the display of inter-annotator agreement for proposed relations, as well as the confidence of the model in the case of machine learning predictions.

## 6 Conclusions

In this paper, we reported on the construction of a framework for the expansion of scientific databases by assisting human annotation with text mining. The approach involves the compilation of a relation extraction dataset from an existing knowledge base, in this case the BRENDA Enzyme Database, and the development of a machine learning model to identify entity mentions and extract relations between them in research articles. By relying on document-level annotations, the approach is suitable for domains where annotated resources are scarce.

While this paper focused on the specific goal of expanding BRENDA annotations at the strain level, the approach is generalizable to other expert-curated databases that rely on literature annotation. As it stands, the code does not make assumptions about the nature of the entities in the database, and the shape of the classifier layers is directly inferred from the number of entities involved. A planned feature of the annotation interface is the ability to import an ontology specific to the goals of a particular database. With that, different teams should be able to reuse the same interface in different domains, reviewing automatic predictions for those classes and relations (or properties) in the ontology for which a classifier has been trained, as well as adding new annotations for the other classes in the ontology.

A central limitation of the present work lies in the scale and distribution of the entity identifier space. Our dataset comprises more than 6,861 unique identifiers, the majority of which occur only rarely, producing a long-tail extreme multi-label classification problem. As our evaluation shows, while the model achieves reasonable performance on entity class prediction, entity identification suffers from very low F1 scores. This is partly due to the sparsity of positive examples, the indirect inference of negative pairs, and the absence of explicit candidate pruning mechanisms. The observed LRAP scores suggest that the model is nevertheless learning to rank correct entities above incorrect ones, but further work is needed to translate this into usable predictions. Future improvements may include candidate retrieval strategies and per-class calibration or thresholding. In this sense, our current model should be seen as a proof of concept for joint training and representation sharing between subtasks, with the recognition that high-quality identifier-level performance will require additional methodological work.
